# Mitral Transcatheter Edge-to-Edge Repair in INTERMACS 3–4 Profile Patients with Severe Mitral Regurgitation

**DOI:** 10.3390/jcdd11110373

**Published:** 2024-11-19

**Authors:** Simone Frea, Stefano Pidello, Filippo Angelini, Paolo Boretto, Pier Paolo Bocchino, Daniele Melis, Giuseppe Giannino, Elena Cavallone, Francesca Giordana, Sara Rettegno, Carol Gravinese, Giulia De Lio, Guglielmo Gallone, Veronica Dusi, Gianluca Alunni, Antonio Montefusco, Fabrizio D’Ascenzo, Massimo Boffini, Claudia Raineri, Mauro Rinaldi, Gaetano Maria De Ferrari

**Affiliations:** 1 Cardiovascular and Thoracic Department, Division of Cardiology, Città della Salute e della Scienza Hospital, 10126 Turin, Italy; frea.simone@gmail.com (S.F.); stefano.pidello@gmail.com (S.P.); paoloboretto14@gmail.com (P.B.); pierpaolo1991@gmail.com (P.P.B.); dany.melis94@gmail.com (D.M.); giuseppe.giannino94@gmail.com (G.G.); elena.cavallone.to@gmail.com (E.C.); francy.giordana@gmail.com (F.G.); sara.rettegno@gmail.com (S.R.); gravinesecarol@gmail.com (C.G.); giulia.delio@edu.unito.it (G.D.L.); guglielmo.gallone@gmail.com (G.G.); veronica.dusi@gmail.com (V.D.); a.gianluca1@virgilio.it (G.A.); anto.montefusco@gmail.com (A.M.); fabrizio.dascenzo@gmail.com (F.D.); cloud.raineri@gmail.com (C.R.); gaetanomaria.deferrari@unito.it (G.M.D.F.); 2 Department of Medical Sciences, Division of Cardiology, University of Turin, 10126 Turin, Italy; massimo.boffini@unito.it (M.B.); mauro.rinaldi@unito.it (M.R.); 3 Division of Cardiac Surgery, Città della Salute e della Scienza Hospital, 10126 Turin, Italy

**Keywords:** TEER, advanced heart failure, inotropes, LVAD, heart transplantation

## Abstract

Background: Heart transplantation and left ventricular assist device (LVAD) implementation are effective treatments for advanced heart failure (HF), although their use is limited by organ availability and the high incidence of adverse events. The efficacy of mitral transcatheter edge-to-edge repair (TEER) as a bridge to transplantation or as a destination therapy in advanced HF is still debated. Methods: A total of 63 patients with INTERMACS class 3 or 4 with contraindications for LVAD and severe functional mitral regurgitation (FMR) were evaluated for TEER implantation eligibility. The primary endpoint was a composite of death, urgent heart transplantation and LVAD implantation at 12 months. Results: A total of 36 patients underwent TEER, while 27 patients received optimal medical therapy (MT) alone. In the intervention group, 35 patients (97%) were discharged alive. In the MT group, two in-hospital deaths occurred, two patients underwent urgent heart transplantation, and three patients were discharged on inotropes. At the 12-month follow-up, the incidence of the primary endpoint occurring was lower in the TEER group (25% vs. 70%, HR 0.25, 95% CI 0.11–0.60, *p* < 0.01) and the tolerance to neurohormonal therapy was higher (53% vs. 30%, *p* = 0.03). Conclusions: In advanced HF patients with INTERMACS profile 3 or 4 and severe FMR, TEER on top of optimal MT was associated with a lower incidence of death, urgent heart transplantation or LVAD implantation at 12 months compared to optimal MT alone.

## 1. Introduction

Advanced chronic heart failure (HF) [1] is characterized by recurrent hospitalizations [2], poor quality of life and reduced life expectancy. Heart transplantation and left ventricular assist device (LVAD) implantation are viable options for only a minority of patients. Alternative therapies aimed at improving symptoms are particularly appealing in Interagency Registry for Mechanically Assisted Circulatory Support (INTERMACS) profile 3 or 4 patients with comorbidities precluding heart transplantation and LVAD implantation [3,4,5].

Since the EVEREST I study [6], mitral transcatheter edge-to-edge repair (TEER) has proven to be safe and effective in reducing mitral regurgitation (MR) [7,8]. Thereafter, a few registries [9,10,11] and meta-analyses [12,13] have confirmed the feasibility and the usefulness of this procedure in symptomatic patients at high surgical risk in terms of the reduction of re-hospitalizations, improvement of symptoms and increased survival [14]. However, the outcomes of TEER have rarely been reported in very-high-risk settings, namely bridge to heart transplantation [15], rescue therapy in cardiogenic shock [16] or during extracorporeal membrane oxygenation (ECMO) support [17].

The aim of this prospective study was to evaluate the clinical impact of TEER as a bridge to transplantation or as a destination therapy in advanced HF patients with INTERMACS profiles 3 to 4 with contraindications for LVAD [18].

## 2. Materials and Methods

### 2.1. Study Participants

This study was a prospective single-center study that screened consecutive patients with INTERMACS profile 3 or 4 who were admitted for acute decompensated advanced HF in the Intensive Cardiac Care Unit of the Division of Cardiology at the Città della Salute e della Scienza University Hospital in Turin from February 2012 to February 2019. This study was approved by the institutional review board, and all the patients provided written informed consent.

The inclusion criteria were aged ≥ 18 years, acute decompensation of advanced chronic HF, INTERMACS profile 3 or 4, left ventricle ejection fraction (EF) < 30%, severe functional MR as defined by an effective regurgitant orifice area (EROA) ≥ 40 mm^2^, and being considered for TEER as a treatment option by the advanced HF team. TEER was indicated as a destination therapy in patients with contraindications for LVAD [19] or as a bridge to transplantation in patients listed for heart transplantation if one of the following conditions was fulfilled:

- Patient with INTERMACS profile 3 and absolute contraindications for LVAD
- Patient with INTERMACS profile 4 and relative contraindications for LVAD
- Patient with INTERMACS profile 4 and listed for heart transplantation without pharmacologically irreversible pulmonary hypertension

The absolute contraindications for LVAD were severe right ventricle dysfunction, contraindications for anticoagulation, lack of social support, refusal of implantation, poor adherence to medical treatment, severe comorbidities limiting life expectancy to less than 2 years, porcelain aorta or the presence of 2 or more relative contraindications. The relative contraindications were malnutrition, high bleeding risk, moderate to severe aortic regurgitation, active systemic infection, severe peripheral vascular disease, and severe chronic kidney disease. Both the MR and INTERMACS profiles were assessed after achievement of optimal hemodynamic compensation.

INTERMACS profile 3 was defined as the failure of two attempts to wean the patient from inotropic agents indicated by the occurrence of worsening HF signs and symptoms and/or progressive end-organ hypoperfusion. The additional inclusion criteria for INTERMACS 4 profile were at least one hospitalization in the last 6 months for acute HF with hypoperfusion [20] and the need for inotropes during the index hospitalization (vasoactive inotropic score ≥ 3 micrograms/kg/min for at least 24 h).

The exclusion criteria were complex adult congenital heart disease, primary valvular disease, and primary right-sided disease.

### 2.2. Study Design

Hemodynamically stable patients who met the inclusion criteria underwent transesophageal echocardiography to evaluate their anatomical eligibility for TEER implantation [6]. Hemodynamic stability was defined as the presence of a dry and warm profile, with no increase in the dose of intravenous diuretics and inotropes during the preceding 24 hours. Anatomically eligible patients were enrolled in the TEER treatment group and underwent TEER during the index hospitalization, while ineligible patients were included in the control group. The INTERMACS 3 patients in the control group underwent repeated levosimendan [21,22] or continuous infusion of dobutamine treatment to maintain patient stability and organ perfusion.

After the index hospitalization, the patients were followed and treated by the advanced HF team according to best medical practice. All the patients were followed for at least 12 months.

### 2.3. Endpoints

The primary endpoint was the composite of all-cause death, urgent heart transplantation and LVAD implantation at 12 months, and the individual components of the composite endpoint were also evaluated. The secondary endpoints were a composite of death or hospitalization for HF at 12 months and sustained tolerance to angiotensin-converting enzyme inhibitor (ACE-i), angiotensin receptor blocker (ARB) or angiotensin receptor neprilysin inhibitor (ARNI) therapy after 12 months, which was defined as the capability to receive ACEi/ARB/ARNI for at least 9 out of 12 months.

### 2.4. Clinical Evaluation and Laboratory Tests

At enrollment, demographic data were collected. The HF treatment during the previous month was recorded; in particular, ACEi/ARB/ARNI tolerance was carefully investigated. Clinical data and laboratory test results were collected both at admission and after hemodynamic stability was reached. The clinical examination included careful assessment of congestion and hypoperfusion (according to the cold-modified model) [23]. The laboratory tests included markers of end-organ damage such as creatinine, blood urea nitrogen, serum sodium, aspartate aminotransferase alanine aminotransferase bilirubin, ammonia [24], high-sensitivity troponin T and N-terminal pro-B-type natriuretic peptide (NT-proBNP). The INTERMACS profile was evaluated at admission and reassessed when optimal compensation was achieved. The expected 1-year mortality rate was estimated using the Seattle Heart Failure Model (SHFM) [25].

### 2.5. Echocardiogram

The echocardiogram examination was performed according to the American Society of Echocardiography guidelines [26]. The left ventricular (LV) chamber size and systolic and diastolic function (with EF evaluation and E/E’ ratio) were measured [27]. Severe mitral regurgitation was defined as an EROA ≥ 40 mm^2^. The basal right ventricular (RV) end-diastolic diameter and tricuspid annular plane systolic excursion were measured. The tricuspid regurgitation pressure gradient (TR gradient) and the systolic pulmonary artery pressure were estimated by the tricuspid regurgitation peak velocity and right atrial pressure (RAP). The RAP was estimated by the inferior vena cava diameter and collapse with sniff and the tricuspid E/e’ ratio and hepatic vein flow analysis in doubtful cases [28]. Finally, the RV contraction pressure index was calculated as TAPSE × TR gradient [29].

### 2.6. Right Heart Catheterization

Right heart catheterization was performed in all the patients. The primary indications were the evaluation before heart transplantation and/or LVAD implantation. Right heart catheterization was performed according to a pre-specified methodological protocol [30]. The pulmonary artery pulsatility index (PAPi) and the ratio of the RAP over the pulmonary capillary wedge pressure (WP) were derived to assess the RV function [31].

### 2.7. TEER Procedure

TEER was performed as previously described [32]. All the procedures were guided by transesophageal echocardiography, with 3D multiplanar reconstruction when available to enhance the visualization and accuracy [33]. The number of clips implanted was left to the operator’s discretion. Patients underwent pre- or peri-procedural levosimendan infusion at the caring cardiologist’s discretion.

### 2.8. Statistical Analysis

The categorical variables are reported as absolute numbers and percentages, while the continuous variables are reported as means and standard deviations or medians and interquartile ranges as appropriate. The correlations between variables, endpoints and treatments were tested in cross-tabulation tables using Fisher’s exact test for categorical and Student’s t-test for continuous variables. The survival rates up to 12 months are presented as Kaplan–Meier curves. To evaluate the independent association of TEER with the primary endpoint, a multivariate analysis was performed. All the significant variables in the univariate analysis by primary endpoint were included in the model. A two-sided *p*-value < 0.05 was considered statistically significant; all the analyses were performed with SPSS 26.0 (IBM Corp., Armonk, NY, USA).

## 3. Results

**Figure 1:**
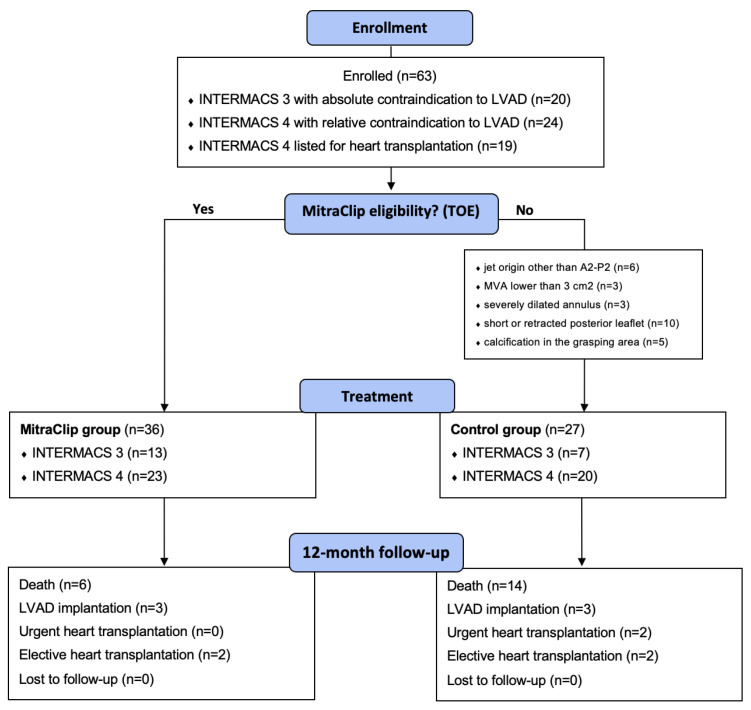
Study flow chart.

Sixty-three patients were included in this prospective analysis. As shown in Figure 1, 20 (32%) patients had an INTERMACS 3 profile with absolute contraindications for LVAD implantation (mainly due to severe right ventricular dysfunction), 24 (38%) patients had an INTERMACS 4 profile and relative contraindications for LVAD (mainly due to severe chronic kidney disease or high bleeding risk), while 19 (30%) patients had an INTERMACS 4 profile and were listed for heart transplantation.

Following the transesophageal echocardiogram, 36 (57%) patients were considered anatomically eligible for TEER and underwent TEER implantation, while 27 (43%) patients were not eligible and represented the control group. As reported in Figure 1, the causes of ineligibility were a short or retracted posterior leaflet (n = 10), jet origin other than A2-P2 (n = 6), calcifications in the grasping area (n = 5), a mitral valve area < 3 cm^2^ (n = 3) and severely dilated annulus (n = 3).

The baseline characteristics of the two groups are shown in Table 1. The preoperative clinical characteristics were not significantly different between the groups except for age, which was significantly higher among the patients undergoing TEER. Specifically, the inotropes dependency and echocardiographic and hemodynamic variables were similar between the study groups.

In the TEER group, a mean of 1.6 ± 0.6 clips per patient (MitraClip NT) were implanted. Procedural success was reached in all the patients. One patient experienced intraprocedural pericardial effusion, which required pericardiocentesis, and another had perioperative intracranial hemorrhage. MR was reduced to a mild degree in all but one case (97%), and after 12 months, MR relapsed to a moderate or higher degree in four (11%) patients. A total of 35 (97%) patients were discharged alive, while among the 27 medically treated patients, in-hospital death occurred in 3 (11%) patients.

As shown in Table 2, at 12 months, 28 patients (44%) met the primary endpoint. Twenty patients (32%) died, two (3%) underwent urgent heart transplantation and six (10%) underwent LVAD implantation. A total of 4 patients (6%) received elective heart transplantation, while 31 patients (49%) were hospitalized for HF.

Patients who died within the 12-month follow-up period presented similar characteristics compared to patients who survived (Table S1), except for lower sodium levels (131 ± 5 vs. 134 ± 5, *p* = 0.02) and a worse right ventricle function (TAPSE 13 ± 4 vs. 17 ± 5; RVCPI 468 ± 200 vs. 711 ± 312), while the difference in the Seattle Heart Failure Model was not significant (33 ± 14 vs. 27 ± 14, *p* = 0.1).

Among the six patients who underwent LVAD implantation, only one patient originally presented an absolute contraindication consisting of two coexistent relative contraindications, one of which (malnutrition) was gradually resolved. One patient presented a relative contraindication and was initially excluded from LVAD but progressively slid from INTERMACS 4 to 3 and was therefore implanted. The remaining four patients were listed for heart transplantation, had no contraindications for LVAD and were implanted after worsening heart failure.

At 12 months, the patients in the control group more likely met the composite primary endpoint or died compared to the patients in the treatment group (19 [70%] vs. 9 [25%], *p* < 0.01, and 14 [52%] vs. 6 [17%], *p* < 0.01, respectively); no significant difference was found regarding urgent heart transplantation and LVAD implantation between the two study groups at 12 months.

**Figure 2:**
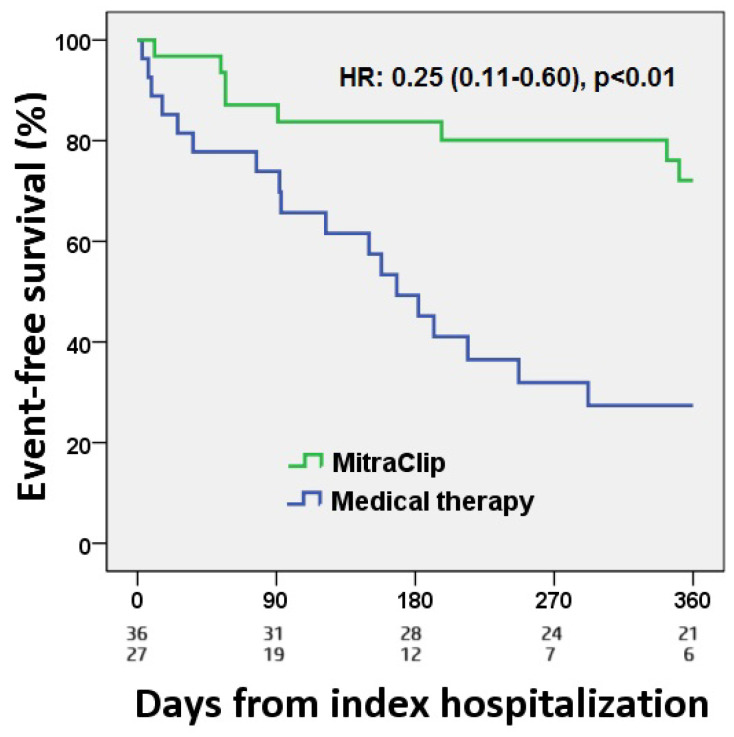
Kaplan–Meier plot by primary endpoint.

The event-free survival at 12 months was significantly higher in the TEER group than in the control group (75% vs. 30%, HR 0.25, CI 95% 0.11–0.60, *p* < 0.01; Figure 2).

TEER was associated with a significantly better composite primary outcome independently from the INTERMACS profile (INTERMACS profile 3: HR 0.19, 95% CI 0.05–0.66, *p* < 0.01; INTERMACS profile 4: HR 0.32, 95% CI 0.13–0.81, *p* = 0.02).

After 12 months, a composite endpoint of death and hospitalization for HF occurred more often in the control group than in the TEER group (85% vs. 53%, *p* = 0.01).

After 12 months, 19 patients (53%) in the TEER group and 8 (30%) in the control group showed sustained tolerance to ACE-i/ARB/ARNI (*p* = 0.03). Among the patients with ACE-i/ARB/ARNI intolerance at enrolment (47 patients), the initiation of ACE-i/ARB/ARNI therapy was better tolerated in the TEER group than the control group (14/27 vs. 1/20, 52% vs. 5%, *p* < 0.01). Two patients underwent elective heart transplantation in the TEER group at 12 months.

In the TEER group, all 13 INTERMACS profile 3 patients were successfully weaned from inotropes after TEER within the index hospitalization and discharged alive. During follow-up, 10 of them (77%) improved to INTERMACS profile 5 or higher, 2 (15%) patients died, and 1 (8%) patient was re-hospitalized for HF after two months.

Among the seven INTERMACS profile 3 patients in the control group, two (29%) in-hospital deaths occurred, two (29%) patients underwent urgent heart transplantation, two (29%) patients were discharged with a repeated levosimendan infusion schedule and one (14%) patient received palliative care with continuous dobutamine infusion.

## 4. Discussion

This prospective monocentric study showed that TEER in patients classified as INTERMACS profile 3 or 4 is feasible and associated with a lower incidence of the primary composite endpoint, lower mortality and higher tolerance to ACE-i/ARB/ARNI initiation than the control group.

Mitral TEER has already been reported in advanced HF patients [34,35,36], and several scores have been proposed to assess the risk of outcomes in this population [37,38,39], but sparse data exist on TEER as a bridge to heart transplantation or LVAD implantation [10,40] and the therapeutic options in advanced heart failure are limited [41,42].

The International MitraBridge registry included 153 advanced HF patients (median age 59 years, LVEF 26.9 ± 7.7%) with significant secondary MR, who were potential candidates for heart transplantation and were treated with TEER as a bridge-to-transplant strategy. At 24 months, the freedom from the primary composite endpoint (all-cause death, first hospitalization for HF, urgent heart transplantation or LVAD implantation) was 47%, with an annualized rate of HF re-hospitalization per patient-year of 44%. Interestingly, 32 patients (22.5%) no longer required heart transplantation after mTEER due to significant clinical improvement [43].

In 2023, Price TJ et al. published the results of a retrospective case series on 11 patients with severe mitral regurgitation on chronic milrinone (namely INTERMACS profile 3); after mTEER, 73% of patients were effectively weaned off inotropes [44].

This is the first prospective study focusing on INTERMACS profile 3 or 4 patients. In our study, mitral TEER proved to be safe, with a rate of intra-procedural complications in line with the literature [45], and it was effective in reducing MR even in this setting.

In our study, the TEER group showed a lower incidence of the primary composite endpoint as well as a lower incidence of all-cause death or re-hospitalization for heart failure at 12 months. Also, after TEER, all the INTERMACS 3 profile patients were successfully weaned off inotropes. These favorable results are of interest since the prognostic impact of MR correction is often questioned in severely dilated and dysfunctional left ventricles. On the other hand, it has recently been suggested that patients are more likely to benefit from TEER if the MR grade is disproportionate to the LV end-diastolic volume [45] and if the right ventricular function assessed by PAPi is not severely compromised [30]. In our cohort, the mean LV end-diastolic volume was 248 ml and the mean EROA was 49 mm^2^, consistent with the definition of disproportionate MR. Moreover, the mean PAPi was 5.1, demonstrating a good RV function in our population. Accordingly, our results suggest that TEER is feasible and may be associated with better outcomes than lone medical treatment in selected patients with advanced HF.

Despite the short-term follow-up and the observational and non-randomized design of our study, these results are encouraging since improved hemodynamic stability, increased hospitalization-free survival and preservation of end-organ function are pivotal treatment targets of both a bridge-to-transplant strategy and a destination therapy in patients with no indication for heart transplantation or LVAD. On the other hand, it should be emphasized that, though mortality was quite low in the TEER group, the re-hospitalization rate remained high in both treatment groups. This emphasizes that the benefit of TEER in advanced HF is lower than in the setting of less advanced disease. Accordingly, despite TEER being feasible in advanced HF patients, the heart team should not overestimate the benefit of TEER and should not postpone LVAD implantation if mechanical circulatory support is indicated and feasible.

Furthermore, the tolerance of neurohormonal antagonists improved after TEER. This result may depend on an increased cardiac output after TEER. As hemodynamic–renal limitation of neurohormonal therapy is associated with a worse outcome, while a restored tolerance may be associated with an additional positive effect on the prognosis [46].

We must acknowledge several limitations. First, this was an observational study. Since the patients were assigned to the treatment group according to the anatomical feasibility of TEER, the causality between TEER and the occurrence of adverse events cannot be assumed. Particularly, even in the absence of significant differences between the two groups, the differences in mitral morphology may reflect different LV function and prognosis. Moreover, since this was a single-center study, our results may not be completely reproduced in centers with different clinical practice. Finally, since the sample size was low, these results should be confirmed in larger populations.

## 5. Conclusions

In advanced HF patients with INTERMACS profile 3 or 4 and severe MR, mitral TEER was associated with better outcomes compared to medical treatment. Mitral TEER was also associated with successful weaning off inotropes and with greater tolerance of neurohormonal antagonists.

## Figures and Tables

**Table 1:** Baseline characteristics of study population.

	TEER (n = 36)	MT (n = 27)	*p* Value
Age (years)	65 ±10	60 ± 12	0.04
Male sex (%)	31 (86)	21 (78)	0.2
Ischemic etiology (%)	22 (61)	13 (48)	0.2
Systolic blood pressure (mmHg)	98 ± 11	99 ± 15	0.8
INTERMACS profile 3 (%) INTERMACS profile 4 (%)	13 (36) 23 (64)	7 (26) 20 (74)	0.2
Hospitalization for HF in the previous 6 months (n/6 months)	1.7 ± 0.8	1.8 ± 1.2	0.8
Seattle Heart Failure Model (1-year estimated survival, %)	27 ± 15	30 ± 14	0.4
Sodium (mEq/L)	134 ± 4	133 ± 7	0.5
NT-proBNP (pg/ml)	9679 (7102–14,183)	10,570 (4531–14,968)	0.4
Creatinine (mg/dl)	1.5 ± 0.5	1.7 ± 0.8	0.3
Hemoglobin (mg/dl)	11.8 ± 2.1	12.3 ± 2.1	0.4
Beta-blockers (%)	32 (90)	23 (85)	0.4
ACE-i/ARB/ARNI (%)	9 (25)	12 (44)	0.1
Echocardiography			
LVEDD (mm)	73 ± 21	72 ± 13	0.7
LVEDV (ml)	241 ± 98	258 ± 93	0.5
Left ventricle ejection fraction (%)	21 ± 3	21 ± 4	0.9
EROA (mm^2^)	51 ± 13	51 ± 19	1.0
Coaptation depth (mm)	12 ± 2	12 ± 2	0.8
TAPSE (mm)	17 ± 5	15 ± 5	0.1
RVCPI (mm·mmHg)	680 ± 317	608 ± 274	0.4
Right heart catheterization			
Cardiac index (l/min/m^2^)	2.0 ± 0.6	2.0 ± 0.5	0.9
Wedge pressure (mmHg)	23 ± 8	22 ± 9	0.7
sPAP (mmHg)	48 ± 15	47 ± 14	0.9
Pulmonary artery pulsatility index	5.8 ± 8.3	4.6 ± 4.5	0.5
Right atrial pressure (mmHg)	9 ± 6	8 ± 5	0.8
Right atrial/wedge pressure ratio	0.36 ± 0.18	0.37 ± 0.12	0.9

ACE-i/ARB/ARNI: angiotensin-converting enzyme inhibitor or angiotensin receptor blocker or angiotensin receptor neprilysin inhibitor; EROA: effective regurgitant orifice area; LVEDD: left ventricle left diastolic diameter; LVEDV: left ventricle left diastolic volume; MT: medical therapy; RVCPI: right ventricle contraction pressure index; sPAP: systolic pulmonary artery pressure; TAPSE: tricuspid annular plane systolic excursion; TEER: transcatheter edge-to-edge repair.

**Table 2:** Study endpoints.

	TEER (n = 36)	MT (n = 27)	HR (CI 95%)	*p* Value
Primary endpoint				
Death or urgent heart transplantation or LVAD implantation at 12 months	9 (25%)	19 (70%)	0.25 (0.11–0.60)	<0.01
Death	6 (17%)	14 (52%)	0.23 (0.09–0.61)	<0.01
Urgent heart transplantation	0 (0%)	2 (7%)	0.29 (0.05–1.63)	0.2
LVAD implantation	3 (8%)	3 (11%)	0.62 (0.12–3.15)	0.4
Secondary endpoints				
Death or re-hospitalization for heart failure at 12 months	19 (53%)	23 (85%)	0.42 (0.23–0.79)	<0.01
Death	6 (17%)	14 (52%)	0.23 (0.09–0.61)	<0.01
Re-hospitalization for heart failure	16 (44%)	15 (55%)	0.73 (0.33–1.63)	0.4
Sustained tolerance to ACE-i/ARB/ARNI	19 (53%)	8 (30%)	-	0.03

ACE-i/ARB/ARNI: angiotensin-converting enzyme inhibitor or angiotensin receptor blocker or angiotensin receptor neprilysin inhibitor; LVAD: left ventricular assist device; TEER: transcatheter edge-to-edge repair; MT: medical therapy.

## Data Availability

The raw data supporting the conclusions of this article will be made available by the authors on request.

## Supplementary Materials

The following supporting information can be downloaded at: https://www.mdpi.com/article/10.3390/jcdd11110373/s1, Table S1. Baseline characteristics stratified by death at 12 months.
