# Supplementary material for: Mitral Transcatheter Edge-to-Edge Repair in INTERMACS 3–4 Profile Patients with Severe Mitral Regurgitation
Source: J Cardiovasc Dev Dis. 2024 Nov 19;11(11):373. doi: 10.3390/jcdd11110373 (PMC11595302; doi:10.3390/jcdd11110373)
Supplement: Supplementary file 1 [file jcdd-11-00373-s001.zip › jcdd-3279513-supplementary.pdf]

Supplementary material

Table S1. Baseline characteristics stratified by death at 12 months

	Deceased (n=20)	Survived (n=43)	p value
Age (years)	64 ± 8	62 ± 12	0.4
Male sex (%)	14 (70)	38 (88)	0.1
Ischemic Etiology (%)	10 (50)	23 (53)	0.6
Systolic Blood Pressure (mmHg)	95 ± 8	99 ± 14	0.3
INTERMACS profile 3 (%)<br>INTERMACS profile 4 (%)	6 (30)<br>14 (70)	14 (33)<br>29 (67)	0.8
Hospitalization for HF in the previous 6 months (n/6 months)	2.0 ± 1.2	1.6 ± 0.8	0.1
Seattle Heart Failure Model (1-year estimated survival, %)	33 ± 14	27 ± 14	0.1
Sodium (mEq/L)	131 ± 5	134 ± 5	0.02
NTproBNP (pg/ml)	10619 [6598-16313]	8719 [4765-12677]	0.4
Creatinine (mg/dl)	1,8 ± 0,6	1,5 ± 0.8	0.08
Beta-Blockers (%)	8 (40)	26 (60)	0.4
ACE-i/ARB/ARNI (%)	17 (85)	38 (88)	0.9
**Echocardiography**			
LVEDD (mm)	70 ± 10	73 ± 20	0.5
LVEDV (ml)	263 ± 97	241 ± 90	0.4
Ejection Fraction (%)	20 ± 3	21 ± 4	0.2
EROA (mm^2)	55 ± 22	47 ± 18	0.3
Coaptation depth (mm)	11 ± 2	12 ± 3	0.4
TAPSE (mm)	13 ± 4	17 ± 5	0.01
RVCPI (mm·mmHg)	468 ± 200	711 ± 312	0.01
**Right heart catheterization**			

Cardiac index (l/min/m2)	2.1 ± 0.6	2.0 ± 0.6	0.7
Pulmonary Artery Wedge pressure (mmHg)	25 ± 8	22 ± 9	0.3
Systolic pulmonary artery pressure (mmHg)	53 ± 12	45 ± 15	0.1
Pulmonary Artery Pulsatility Index	4.7 ± 5.1	5.4 ± 7.4	0.7
Right atrial pressure (mmHg)	11 ± 6	8 ± 5	0.1
Right atrial / Wedge pressure ratio	0.36 ± 0.41	0.13 ± 0.35	0.2

Legend. ACE-i/ARB/ARNI: angiotensin-converting enzyme inhibitors or angiotensin receptor blocker or angiotensin receptor neprilysin inhibitor; EROA: effective regurgitant orifice area; LVEDD: left ventricle left diastolic diameter; LVEDV: left ventricle left diastolic volume; MT: medical therpay; RVCPI: right ventricle contraction pressure index; sPAP: systolic pulmonary artery pressure; TAPSE: tricuspid annular plane systolic excursion; TEER: transcatheter edge-to-edge repair.
